# A Simple Cerium Coating Strategy for Titanium Oxide Nanotubes’ Bioactivity Enhancement

**DOI:** 10.3390/nano11020445

**Published:** 2021-02-10

**Authors:** Serena De Santis, Giovanni Sotgiu, Francesco Porcelli, Martina Marsotto, Giovanna Iucci, Monica Orsini

**Affiliations:** 1 Department of Engineering, Roma Tre University, Via Vito Volterra 62, 00146 Rome, Italy; giovanni.sotgiu@uniroma3.it (G.S.); francesco.porcelli@uniroma3.it (F.P.); monica.orsini@uniroma3.it (M.O.); 2 Department of Science, Roma Tre University, Via della Vasca Navale 79, 00146 Rome, Italy; martina.marsotto@uniroma3.it (M.M.); giovanna.iucci@uniroma3.it (G.I.)

**Keywords:** titanium, cerium oxide, nanotubes, bioactivity

## Abstract

Despite the well-known favorable chemical and mechanical properties of titanium-based materials for orthopedic and dental applications, poor osseointegration of the implants, bacteria adhesion, and excessive inflammatory response from the host remain major problems to be solved. Here, the antioxidant and anti-inflammatory enzyme-like abilities of ceria (CeO_x_) were coupled to the advantageous features of titanium nanotubes (TiNTs). Cost-effective and fast methods, such as electrochemical anodization and drop casting, were used to build active surfaces with enhanced bioactivity. Surface composition, electrochemical response, and in vitro ability to induce hydroxyapatite (HA) precipitation were evaluated. The amount of cerium in the coating did not significantly affect wettability, yet a growing ability to induce early HA precipitation from simulated body fluid (SBF) was observed as the oxide content at the surface increased. The presence of 4%wt CeO_x_ was also able to stimulate rapid HA maturation in a (poorly) crystalline form, indicating an interesting potential to induce rapid in vivo osseointegration process.

## 1. Introduction

Titanium (Ti)-based materials are widely used for orthopedic and dental implants thanks to their favorable mechanical properties, corrosion resistance in body fluids, and excellent biocompatibility [1]. However, titanium-based implants are often reported to be subject to long term complications, mostly related to loosening of the implant–host interface and susceptibility of the implant to bacterial infections [2]. Poor osseointegration is the basis of both phenomena. As a bio-inert material, Ti is not able to actively interact with the surrounding environment and to favor a satisfactory cell adhesion, which are instead key points for the formation of the structural and functional direct connection between the living bone and the implant surface needed to ensure long-term stability [3]. Great efforts have been paid to improve bone–implant contact, such as changing the substrate surface topography [4,5], chemically modifying the surface layer [6,7], and coating the implant with bioactive materials [8,9]. Fabricating a TiO_2_ nanotube array (TiNT) by means of direct electrochemical anodic oxidation of the titanium substrate is one of the most promising approaches. This porous oxide layer, tightly bonded to the bulk material, proved able to establish beneficial interactions with osteogenic cell types, in terms of adhesion and differentiation, while limiting bacterial attachment [10,11]. In vitro studies also indicated that TiO_2_ nanostructures could be used as smart delivery systems in implants, thus enabling the possibility of releasing active molecules, such as antibiotics or loaded polymer micelles, directly at the site of implantation [12,13]. Nanotubes’ length, diameter, and composition can be easily controlled by varying the processing parameter, and highly reproducible and homogeneous surfaces can be obtained.

The immune response is a further concern to be considered. Although inflammation is a natural and important stage of the wound healing process in which damaged cells, pathogens, and bacteria are removed from the wound area, prolonged or excessive inflammatory conditions can impair bone remodeling [14]. The oxidative stress which accompanies inflammation, with overproduction of harmful reactive oxygen species (ROS), is known to be responsible for inducing apoptosis of the osteoblastic cell line and inhibiting osteoblast differentiation, finally leading to bone loss [15]. Thus, imparting antioxidant function to the biomaterial is a crucial point to prolong the service life of implants. Therefore, the three key factors prompting the clinical success of an implant are ensuring rapid osseointegration, reducing inflammation around the implant, and preventing bacterial adherence on the implant surface.

Metal elements such as silver (Ag), gold (Au), copper (Cu), zinc (Zn) [16,17] or combinations thereof and with hydroxyapatite [18,19] have been thoroughly examined as titanium-based material enhancers to improve their antibacterial activity. Strontium (Sr) and silicon (Si) have also been considered as essential trace elements in biological processes [20]. More recently, ceria (CeO_2_), one of the most reactive rare-earth metal oxides, has received growing attention in biomedical applications due to its unique physical and chemical properties and reported biocompatibility [21,22,23]. Nanoceria was recently found to have superoxide dismutase, catalase, and oxidase mimetic properties, possessing ROS-scavenging capability comparable to those of biological enzymes [24]. Such characteristic functionalities depend on the presence of mixed valence states (Ce^3+^ and Ce^4+^) and oxygen vacancies that allow this redox couple to rapidly switch between the two forms according to the conditions of the immediate environment [25]. The multiple enzyme activities of CeO_2_ can be exploited to introduce promising antibacterial and anti-inflammatory properties. Ceria has been successfully evaluated in bone regenerative biomaterials as a pure coating [26,27], integrated component [28,29], and nanoparticulated material [30,31], not only confirming its protective capability but also positively influencing the osteogenic activities of bone marrow mesenchymal stem cells (BMSCs) [32]. The aim of the present work was to propose a very simple coating procedure which could eventually be readily transferred into industrial and then clinical applications. With this purpose, a mixed oxide ceria ceramics (CeO_x_) coating onto anodized titanium substrates (TiNT) is realized, employing the drop casting method, a simple and cost-effective deposition method, which allowed for good control over the cerium surface percentage. The effect of varying cerium content is examined, looking for the lowest quantity capable of producing a significant bioactivity enhancement in terms of hydroxyapatite inducing ability.

## 2. Materials and Methods

### 2.1. Samples Preparation

TiO_2_ nanotube arrays (TiNT) were obtained by a one-step anodization process from commercially pure titanium foil (thickness 0.127 mm, 99.7% trace metals basis, Merk Life Science, Milano, Italy). Samples were degreased by ultrasonic cleaning in water/acetone 50:50 and ethanol for 10 min, rinsed with deionized water, and dried under an air stream at ambient temperature. Anodic oxidation was performed under potentiostatic control (15 V, 45 min), using 1 cm × 1.5 cm cleaned Ti foils as working electrodes and a platinum wire as a counter electrode. The two electrodes were placed in an electrolyte consisting of H_2_O/glycerol 40:60 with 0.25 wt% NH_4_F (≥99.99% trace metals basis, Merk Life Science, Milano, Italy). Anodized samples were thoroughly rinsed with deionized (DI) water and air dried. Cerium-coated samples (TiNT_Ce) were obtained by the drop casting technique, repeatedly depositing 20 μL of a 10^−2^ M Ce(NO_3_)_3_∙6H_2_O (99.99% trace metals basis, Merk Life Science, Milano, Italy) solution in ethanol. Each deposition was followed by a 10 min annealing step at 400 °C; in the final step, samples were annealed for 2 h at 400 °C and left to cool to an ambient temperature. Five samples were prepared, named TiNT_Ce_n_, with n being 1, 3, 6, 9, and 12 respectively, based on the number of depositions performed.

### 2.2. Surface Characterization 

Morphological and compositional observations of the samples were obtained by scanning electron microscopy with energy dispersive X-ray spectroscopy (SEM-EDS) using a Zeiss Gemini SIGMA 300 FEG SEM (Jena, Germany) equipped with Bruker EDS (Bruker Italia, Milano, Italy). Micrographs were obtained at 5 kV, while EDS analysis was performed at an accelerating voltage of 20 kV, with a back scattered detector and working distance of 7.5 mm.

XPS analysis was performed with a homemade instrument, consisting of preparation and analysis UHV chambers separated by a gate valve. The analysis chamber is equipped with a six-degree-of freedom manipulator and a 150 mm mean radius hemispherical electron analyzer with a five-lens output system combined with a 16-channel detector, giving a total instrument resolution of 1.0 eV as measured at the Ag 3d_5/2_ core level. Samples were introduced in the preparation chamber and left outgassing overnight at a base pressure of about 10^−8^ Torr, before introduction in the analysis chamber. Typical vacuum pressure in the analysis chamber during measurements was in the 10^−8^–10^−9^ Torr range. The used X-ray radiation was a non-monochromatized Mg Kα(1253.6 eV). The spectra were energy referenced to the Ti 2p_3/2_ signal of titania nanotubes having a binding energy BE = 458.50 eV. Atomic ratio values were calculated from peak intensities. Curve-fitting analysis of the C 1s, N 1s, O 1s, Ti 2p and Ce 3d spectra was performed using Gaussian profiles as fitting functions, after subtraction of a Shirley-type background. Ti2p_3/2,1/2_ and Ce3d_5/2,3/2_ doublets were fitted by using the same Full Width at Half-Maximum (FWHM) for each pair of components of the same core level, a spin–orbit splitting of, respectively, 5.7 and 18.3 eV and branching ratios Ti2p_3/2_/Ti2p_1/2_ =2/1, Ce3d_5/2_/Ce3d_3/2_ =3/2.

### 2.3. Electrochemical Measurements

Corrosion characteristics of the coated samples were investigated using the potentiodynamic polarization test using an AMEL System 5000 workstation (AMEL, Milano, Italy), CorrWare software version 3.5c (Scribner, NE, USA) for the acquisition and CorrView software version 3.5c (Scribner, NE, USA) for the elaboration. Measurements were performed on 1 cm^2^ samples areas at 37 °C using a simulated body fluid (SBF) as an electrolyte in a three-electrode electrochemical cell with the TiNT or TiNT_Ce_n_ sample as the working electrode (WE), a platinum wire as a counter electrode (CE), and Ag/AgCl as the reference electrode. The polarization tests were conducted at a scan rate of 10 mV/s vs. open circuit potential (OCP) in the potential range −400–500 mV.

Electrochemical impedance spectroscopy (EIS) was recorded using a Solartron 1255B Frequency Response Analyzer (AMETEK Scientific Instruments, Milano, Italy). The frequency ranged from 60 kHz to 100 mHz, with an ac amplitude of ±10 mV. Before measurement, samples were immersed in the SBF electrolyte at 37 °C until the open circuit reached a steady-state value. EIS data were analyzed considering equivalent electrical circuits using the ZView fitting program (Scribner, NE, USA). Reproducibility of the results was confirmed by repeating each measurement at least three times.

SBF was prepared according to the standard procedure proposed by Kokubo et al. [33]. The solution composition is reported in Table 1; the final pH was 7.41. All reagents were purchased from Merk Life Science (Milano, Italy) and used as received.

### 2.4. Surface Wettability

Surface wettability was investigated through water contact angle (WCA) measurements carried out using homemade contact angle meter equipment realized with respect to the relative normative (UNI EN 828, UNI 9752, ASTM D-5725-99). Three samples were observed for each condition, depositing four drops (3 μL) on each; the corresponding image was captured with an Olympus Software Imaging System after 20 s stabilization. The contact angle was measured using ImageJ software. 

### 2.5. In Vitro Bioactivity Test

Bioactivity was evaluated upon immersion in SBF, following the standard procedure described by the International Standard ISO 23317:2014 [34]. Samples were placed in sterilized tubes filled with SBF which were then sealed and placed in a thermostatic bath at 37 °C for 8 days. Samples were then thoroughly rinsed with DI water and air dried prior to subsequent analysis. The formation of calcium-phosphate species (CaPs) was assessed by EDS analysis, while identification of their type was obtained by Fourier transformed infrared (FT-IR) microspectroscopy, using a Nicolet iN10 infrared microscope (Thermo Fisher Scientific IT, Milano, Italy) equipped with a mercury-cadmium-telluride (MCT-A) nitrogen-cooled detector in ATR mode. The FTIR spectra were collected in the 4000–650 cm^−1^ range as an average of 64 scans, with 8 cm^−1^ resolution. At least five measurements were acquired from different areas for each sample. OmnicPicta software version 2.03 (Thermo Fischer Scientific, Milano, Italy) was used for post elaboration of the spectra.

## 3. Results and Discussion

The morphology, surface composition, and electrochemical behaviour of TiNT_Ce_n_ (obtained by titanium anodization at 15 V followed by drop casting deposition of a cerium precursor solution and heat treatment at 400 °C) were analysed in detail.

### 3.1. Surface Morphology and Composition

**Figure 1:**
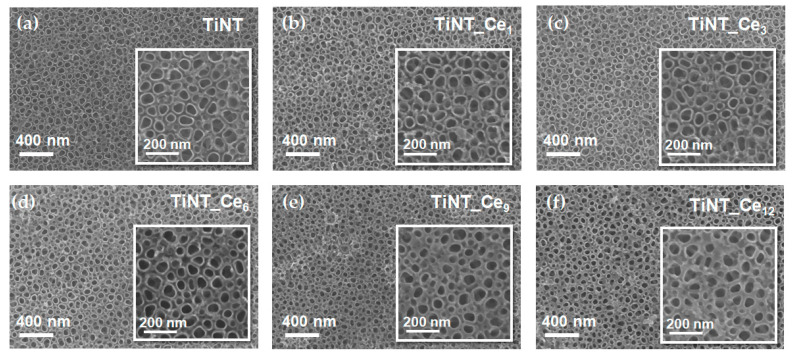
Top surface FE-SEM images of (**a**) TiO_2_ nanotube and (**b**–**f**) cerium-coated TiO_2_ nanotubes with increasing amount of CeO_2_.

The FE-SEM micrographs of the pristine and ceria-coated titanium nanotube samples are given in Figure 1a–f. A uniform and regular distribution of the stacked, unidirectional nanotube array can be observed over the substrate. 

The pristine titania nanotubes are approximately 50 nm in diameter (Figure S1). SEM micrographs suggest that when the Ce(NO_3_)_3_ solution is drop casted onto the surface, cerium ions are mainly deposited on interstices. In fact, as the number of solution depositions increased, the outer diameter of the tubes progressively rose, while the inner diameter was substantially maintained. The cerium oxide layer preferentially occupies the void space between nanotubes, providing a higher nanostructure surface density while preserving the open top nanotubular pattern. The thickening of the nanotube walls is almost negligible for the lower cerium content considered, while it becomes more important for the samples TiNT_Ce_9_ and TiNT_Ce_12_. The original inner size of the nanotubes, which can be finely tuned by choosing the proper parameters during anodization process, is maintained (Figure S1). This is an important feature, since it was demonstrated that TiO_2_ nanotube diameter strongly affects osteogenic cell adhesion, growth, and differentiation [35]. 

Energy dispersive X-ray spectroscopy (EDS) analysis confirms the presence of cerium on all samples surfaces with a percentage varying between 0.8 and 8%, going from TiNT_Ce_1_ to TiNT_Ce_12_, respectively (Figure S2, Table S1). 

**Figure 2:**
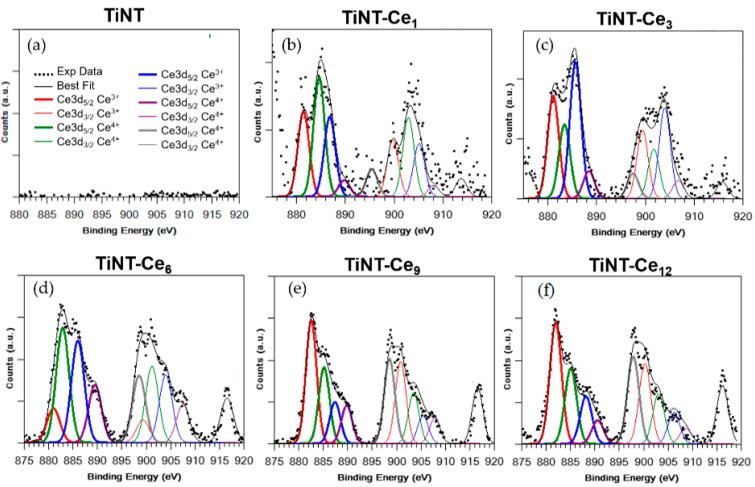
XPS Ce3d fitted spectra for the (**a**) TiO_2_ nanotube (TiNT) and (**b**–**f**) cerium-coated (TiNT_Ce_n_) samples.

X-ray photoelectron spectroscopy (XPS) measurement on TiNT_Ce_n_ samples was carried out at the C 1s, O 1s, Ti 2p and Ce 3d core levels (Figure 2, Figures S3 and S4). Pristine titania nanotubes, anodized in the same conditions, were also measured; the obtained data were used as standard for the interpretation of the signals arising from the coated specimens. Ti2p spectra (Figure S4) are made of one spin-orbit doublet (Ti2p_3/2_, Ti2p_1/2_). The Ti2p_3/2_ signal at 458.5 eV, taken as reference for the Ti2p_3/2−1/2_ spin−orbit pair, is associated to titania nanotubes and so attributed to fully oxidized Ti^4+^ species [36]. It is thus possible to infer that the Ce^3+^ ions of the precursor solution do not interact directly with titanium atoms but rather form Ti-O-Ce bridges, as already observed by other authors [22,37].

By following a peak-fitting procedure, five spin orbit pairs related to Ce3d were individuated (Figure 2), and the resulting components were associated with Ce^3+^ and Ce^4+^ species by comparison with literature data [38]**.** The intensity of the Ce3d_3/2_ signal at higher binding energy, which indicates the amount of ceria in the sample, increased from TiNT_Ce_1_ to TiNT_Ce_12_, as expected. The Ce^3+^/Ce^4+^ ratio can control the enzyme-like behavior of the oxide [27]; this ratio was estimated for every sample considering the total contribution of each species, comparing the area associated with the Ce^3+^ peaks with the total area of the Ce3d spectrum signals. The data showed that an approximately 1:1 ratio mixture of Ce^3+^ and Ce^4+^ was formed by the deposition procedure comprising extensive heat treatment in air. Ce^4+^ coatings exert greater anti-inflammatory effects and proved more efficient at enhancing the osteogenic activities of BMSCs [27]. At the same time, the ability to preserve large amount of reduced cerium atoms is quite important, since in this form, it exhibits the highest ability to bind phosphate species [39]. 

### 3.2. Electrochemical Behavior

**Figure 3:**
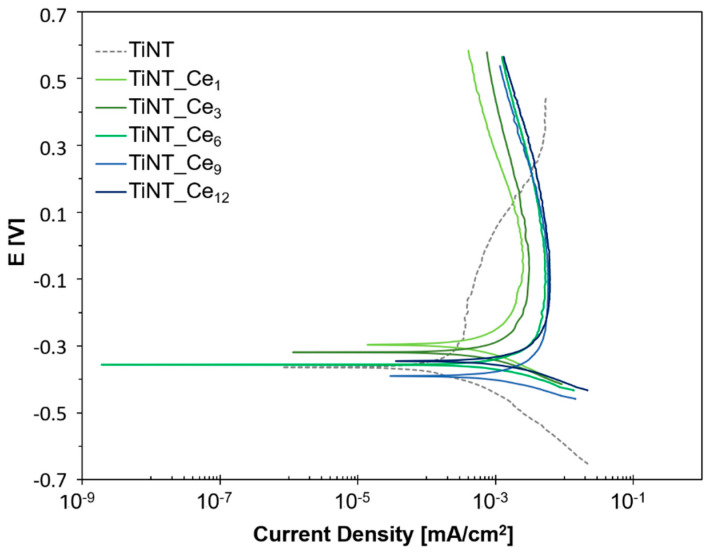
Potentiondynamic polarization curves for TiNT and TiNT_Ce_n_ samples recorded in SBF at 37 °C.

Potentiodynamic polarization studies for the TiNT and cerium-coated samples were performed to gain information about the polarization domains of these surfaces in SBF solution (Figure 3). All samples were immersed in the electrolyte for 2 h to ensure stabilization of OCP before all experiments.

The kinetic parameters obtained by potentiodynamic polarization curve analysis are given in Table 2. 

The I_corr_ and E_corr_ of each sample ranged from 0.57 to 3.93 μA/cm^2^ and from −0.396 to −0.297 V, respectively. The almost corresponding active potential of all the samples suggests very similar corrosion susceptibility in terms of E_corr_. A broad passive domain was quickly formed starting from −0.2 V for all the considered samples, with values of I_pass_ quite similar to those of I_corr_, indicating that the protective behavior is rapidly established. At potential higher than – 0.2 V, the current density of TiNT_Ce_n_ samples showed almost no change with the increase in the potential, except for a constant slight decrease, indicating the formation of a stable oxide layer, which is effective against further corrosion over a wide potential. The uncoated nanotube sample showed an apparent lower I_pass_ value; however, an unstable passive layer is formed in that case. As indicated by the dashed gray line, a continuous increase in the current value is observed until a potential of 0.3 V is reached, at which the current value is higher than for any other TiNT_Ce_n_ sample. These results indicate that cerium-coated samples have good corrosion resistance. The constant behavior at increasing potential suggests that there are no significant mass losses or debris formation from the surface of TiNT_Ce_n_ during the corrosion process, which makes coated samples safer with respect to toxic effects induced by metal leakage.

**Figure 4:**
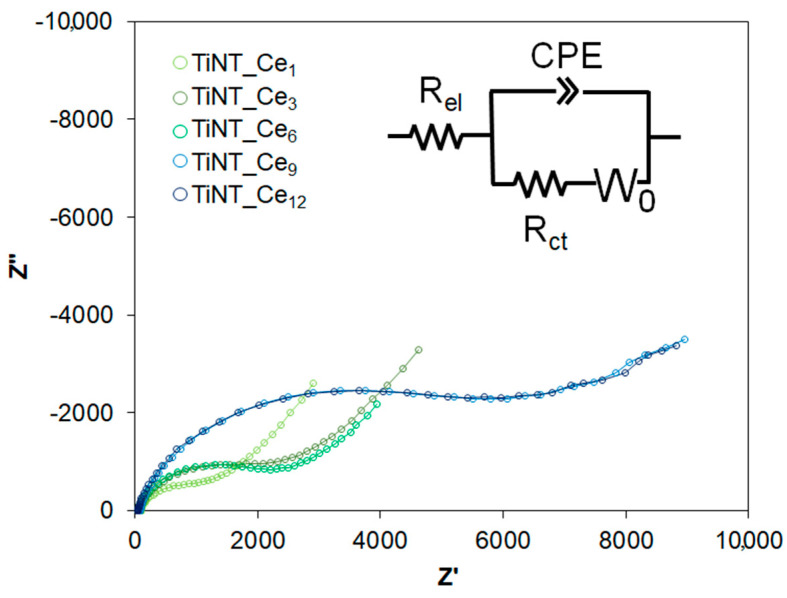
Electrochemical impedance spectroscopy (EIS) spectra of ceria deposited TiNT samples. Empty circles correspond to experimental data, solid lines indicate the corresponding fit. The equivalent circuit used is also represented.

To further characterize the properties of cerium-coated titanium nanotubes, electrochemical impedance spectroscopy (EIS) measurements were carried out (Figure 4). The obtained spectra were interpreted using an equivalent electrical circuit model consisting of a solution resistance (R_el_) in series with a parallel circuit of constant phase element (CPE) and charge transfer resistance (R_ct_). 

The use of a CPE instead of a pure capacitance accounts for the non-homogeneous double layer formed by the nanotubular structure. Because of the diffusional phenomenon observed in the low frequency regime of the Nyquist spectrum, which may be due to the ingress of ionic species within the nanotubular structure of oxide film, an additional Warburg element in series with R_ct_ was also considered. Fit results are summarized in Table 3; χ^2^ values of 10^−3^–10^−4^, obtained for all the parameters, indicate very good agreement between the experimental data and the simulated values.

The charge relaxation coefficient (n) gave information about the non-uniform distribution of charge at the surface of the electrode. The charge distribution characteristics of oxide film were quite similar for all TiNT_Ce_n_ samples, as only minor differences were found in the n values for each sample (Table 3). However, the diminishing value of constant phase element admittance Y_o_ (10.48 to 3.75 μS·s^n^cm^−2^) going from TiNT_Ce_1_ to TiNT_Ce_12_ revealed lower charge dissipation in redox reactions induced by the increase in CeO_x_ content on the surface. This observation suggests that ceria depositions actively contribute to give good barrier characteristics to the surface, exerting a blocking effect toward titanium active sites, as already observed for similar systems obtained by electrodeposition of CeO_x_ on TiNT [22]. The improvement in the barrier properties of surface oxide film was further validated by the higher charge transfer resistance values growing from 0.56 to 3.55 kΩ·cm^2^.

The Warburg element in the circuit represents the semi-infinite linear diffusion through the surface and, in our case, it is also related to the transport of ionic species of the electrolyte trough the nanotubular structure. The Warburg coefficient, σ, can give information about the ease with which ions could reach the substrate to react. A progressive increase in σ values was observed with increasing amounts of ceria on the surface, going from 2.44 kΩ·s^‑1/2^·cm^−2^ for a single cerium ion deposition to 11.40 kΩ·s^−1/2^·cm^−2^ for TiNT_Ce_12_, indicating a lower susceptibility of the surface to ion transport, lowering the risk of corrosion reactions.

### 3.3. Surface Wettability

**Figure 5:**
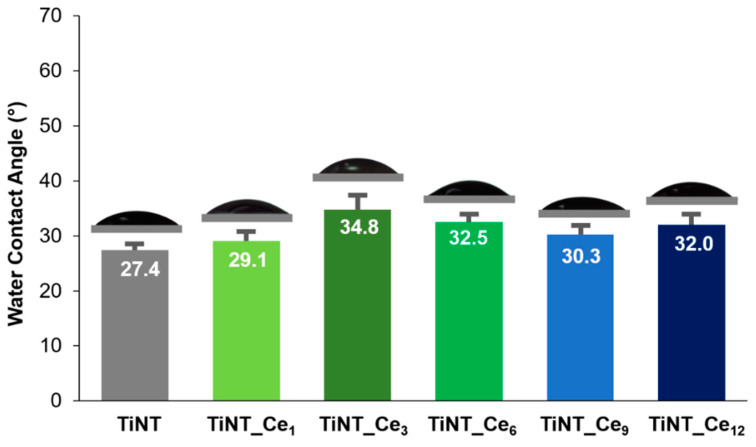
Water contact angle measurements.

Surface wettability plays a major role in determining biomaterial interactions with the physiological surrounding, affecting protein and macromolecules adsorption, cell and bacterial adhesion, and the in vivo rate of osseointegration [40]. Samples’ surface wettability was assessed by measuring their contact angle (θ) with water droplets. The contact angle of TiNT was 22.1° ± 2.2°; cerium-coated samples showed values of θ between 29.1° ± 1.7° and 34.8° ± 2.1° (Figure 5), with a slight decrease in wettability caused by CeO_x_, apparently not proportional to the cerium content. The increase in θ due to cerium was already observed in the literature, and it was connected to a decrease in the active sites on the titanium surface due to the replacement of -OH groups with O-Ce bonds [22]. Nevertheless, the low values of θ indicate a still highly hydrophilic behaviour of the coating which can favour the ion exchange from the body fluids and interactions with proteins. In fact, the good hydrophilicity of nanotube arrays is known to be related to their special structure which is maintained by the coating procedure adopted and seems to exert the predominant effect.

### 3.4. In Vitro Bioactivity

**Figure 6:**
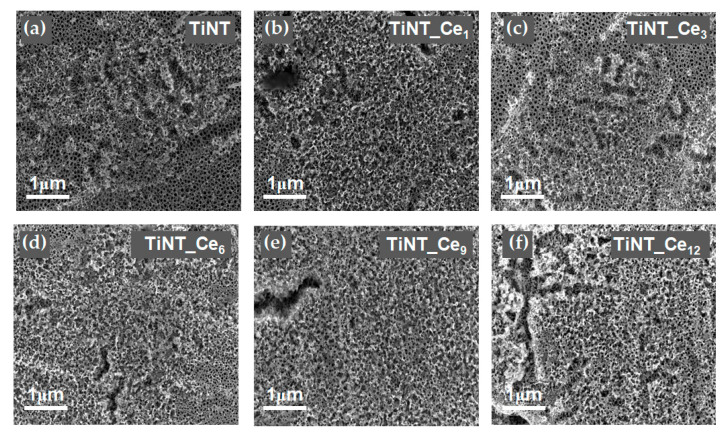
Top surface FE-SEM images of (**a**) TiO2 nanotube and (**b**–**f**) cerium-coated TiO2 nanotubes soaked in SBF 8 days at 37 °C showing the presence of HA precipitate covering the original open top nanostructure.

The integration of biomaterials is crucial to ensure long-term stability of the implant. For a successful bond with tissue to occur, the formation of a layer of biologically active hydroxyapatite (HA) is needed. The mineralization process of calcium phosphates in bone starts from an amorphous calcium-phosphate (CaP) precursor phase which turns into nanocrystalline carbonated HA as a result of an autocatalytic process [41]. It was established that the ability of a material to form a CaP layer in a simulated body fluid, without the support of the protein and cellular components characteristic of the in vivo process, is largely predictive of good bioactive behavior [42]. Thus, the HA forming ability of TiNT_Ce_n_ samples in SBF at 37 °C was investigated. The SEM micrographs after 8 days soaking are shown in Figure 6. Only a low amount of precipitate was found in TiNT micrographs in such a short time (Figure 6a), as already observed in the literature [22]. Instead, all the cerium-coated samples were capable of inducing a significant early apatite-like species growth. In greater detail, a relationship between the cerium surface content and the ability of favoring apatite nucleation is clearly visible (Figure 6b–e). A progressively more compact HA layer is found from TiNT_Ce_1_ to TiNT_Ce_12_, with the latter achieving a complete covering, hiding the underlying nanotubular structure. 

**Figure 7:**
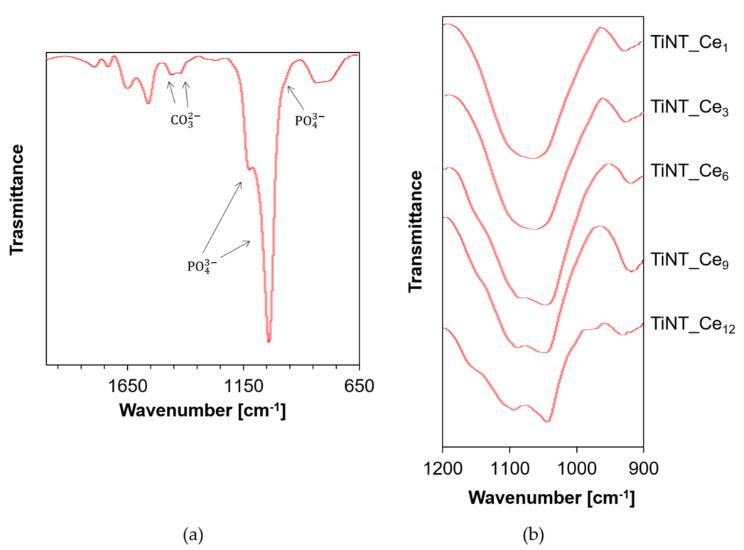
(**a**) ATR-FTIR spectra of TiNT_Ce_12_ after 8 days soaking in SBF at 37 °C (**b**) ν_3_ vibrational mode of PO_4_^3−^ was evidenced for the TiNT_Ce_n_ samples.

The EDS spectra confirmed that Ca and P species are present on the surface, with the elements uniformly distributed along the whole specimen (Figure S5). A Ca/P atomic ratio between 1.42 and 1.57, compatible with non-stoichiometric, calcium-deficient HA, was detected (Table S2). For a deeper investigation of the nature of the Ca/P phase, the ATR-FTIR spectra of TiNT_Ce_n_ were also recorded (Figure 7).

The typical ν_1_ and ν_3_ vibrational mode of phosphate (PO_4_^3−^) bands of apatite were detected at 956, 1055, and 1101 cm^−1^ respectively. The presence of carbonate species was also revealed by the relative bands at 1422 and 1446 cm^−1^. These observations are consistent with carbonate-substituted hydroxyapatite (CHA), with carbonate ions replacing phosphate and/or hydroxyl positions, respectively [43]. CHA is the actual main component of dental and bone tissues in humans and it is known to have an impact on different pathologies [44,45]. It is important to note that CHA is more soluble than HA, thus increasing the local concentration of Ca and P ions and accelerating new bone formation. 

The shape and resolution of the ν_3_ PO_4_^3−^ bands can give qualitative indication about the crystallinity of the HA coating [46]. For lower CeO_x_ content samples, a single broad signal was observed (Figure 7b), indicating that the HA phase is essentially amorphous. Starting from TiNT_Ce_6_, a shoulder is clearly recognizable, becoming more prominent as the amount of CeO_x_ on the surface grows, thus indicating a poorly crystalline type of HA. 

Taken together, the EDS and FTIR results indicate that the presence of cerium on the surface promotes early calcium phosphate species precipitation and, as a function of its concentration, accelerates the maturation of the HA phase, actively favoring the osteointegration process [47]. 

## 4. Conclusions

Cerium-coated titanium nanotubes were prepared by coupling controlled anodic oxidation with a series on drop casting and annealing steps. The homogeneous distribution of the nanotubes and the typical open top topography was preserved even for the highest number of CeO_x_ depositions considered, without altering the original diameters and increasing the homogeneity of the surface. TiNT_Ce_n_ proved to have good corrosion resistance, with cerium exerting a protective action toward the active site on the titanium surface. All the samples showed similar wettability, slightly lower than that of pristine TiNTs. Nevertheless, a growing ability to induce early HA precipitation from SBF was observed as the cerium content of the surface increased. It was also observed that the HA deposited on samples in which the atomic percentage of cerium is higher than 4% (TiNT_Ce_6_) possessed a (poorly) crystalline form, indicative of a more advanced state of maturation and therefore of a potentially more rapid in vivo osseointegration process. Based on the data here discussed, TiNT_Ce_9_ (7% atomic percentage) can be considered as the most favorable, since its electrochemical properties and ability to induce HA maturation are comparable to those of TiNT_Ce_12_ while requiring fewer preparation steps. The influence of cerium content on cell adhesion and viability and its effective antibacterial and anti-inflammatory capabilities are currently under investigation to gain further insight into CeO_x_-based materials properties. Nevertheless, the simple, easily scalable method here proposed stands as an interesting alternative for the improvement of titanium-based implants bioactivity.

## Figures and Tables

**Table 1:** Ion concentration in simulated body fluid (SBF) solution.

Ion	Ion Concentrations (mM)
Na^+^	142.0
K^+^	5.0
Mg^2+^	1.5
Ca^2+^	2.5
Cl^−^	147.8
HCO^3−^	4.2
HPO_4_^2^^−^	1.0
SO_2_^4^^−^	0.5

**Table 2:** Electrochemical parameters of the titanium and cerium-coated samples measured in SBF at 37 °C.

	**E_corr_ (V)**	**I_corr_ (μA)**	**I_pass-0_._2_ (μA)**	**I_pass0_._3_ (μA)**
TiNT	−0.364	0.57	0.37	4.87
TiNT_Ce_1_	−0.297	1.70	1.83	0.95
TiNT_Ce_3_	−0.320	1.36	2.81	1.48
TiNT_Ce_6_	−0.356	3.18	5.72	3.16
TiNT_Ce_9_	−0.396	3.18	5.87	3.18
TiNT_Ce_12_	−0.345	3.93	5.87	3.26

I_pass-0.2_ and I_pass0.3_: current at a potential of −0.2 and 0.3 V, respectively.

**Table 3:** Electrochemical parameters obtained from the equivalent circuits of the TiNT_Ce_n_ samples by measuring the open circuit potential (OCP) at 37 °C using SBF as the electrolyte.

	**R_el_ (Ω·cm^2^)**	**R_ct_(kΩ·cm^2^)**	**CPE**		**σ (kΩ·s^−1/2^·cm^−2^)**	**χ^2^**
			**Y_o_ (μS·s^n^·cm^−2^)**	**n**		
TiNT_Ce_1_	82.6	0.56	10.48	0.892	2.44	1.00∙10^−3^
TiNT_Ce_3_	77.0	1.32	5.29	0.881	4.64	1.00∙10^−4^
TiNT_Ce_6_	69.6	1.81	5.43	0.876	4.95	1.00∙10^−4^
TiNT_Ce_9_	84.4	2.82	4.76	0.880	10.47	1.00∙10^−4^
TiNT_Ce_12_	70.2	3.55	3.75	0.884	11.40	1.00∙10^−3^

## Data Availability

Not applicable.

## Supplementary Materials

The following are available online at https://www.mdpi.com/2079-4991/11/2/445/s1, Figure S1: Image analysis of the FE-SEM micrographs for TiNT and TiNT_Ce_n_. Panels (a) and (b) show the original SEM output (in gray) partially superimposed onto the binarized image generated for the calculation. Panel c–h report the inner and outer diameter distribution for each sample. Figure S2: FE-SEM micrograph and EDS spectrum relative to the selected area. EDS was performed at 15 kV at the same magnification and corresponding total area for every sample; Figure S3: XPS O1s fitted spectra for (a) TiNT and (b–f) TiNT_Cen samples. Data were fitted with four components of about 530, 531.5, 533, and 534 eV that correspond to O2− of metal oxides, carbonyl oxygens, always present in samples prepared in air, hydroxyl groups (or chemisorbed water) and physisorbed water, respectively, Figure S4: XPS Ti2p fitted spectra for (a) TiNT and (b–f) TiNT_Cen samples, Figure S5: FE-SEM micrograph and EDS spectrum relative to the selected area. EDS was performed at 15 kV at the same magnification and corresponding total area for every sample, Table S1: Elemental composition (in atomic %) of TiNT and TiNT_Cen samples, Table S2: Elemental composition (in atomic %) of TiNT and TiNT_Ce_n_ samples after 8 days soaking in SBF at 37 °C.
