# Supplementary material for: A Simple Cerium Coating Strategy for Titanium Oxide Nanotubes’ Bioactivity Enhancement
Source: Nanomaterials (Basel). 2021 Feb 10;11(2):445. doi: 10.3390/nano11020445 (PMC7916473; doi:10.3390/nano11020445)
Supplement: Supplementary file 1 [file nanomaterials-11-00445-s001.pdf]

nanomaterials

MDPI

*Supplementary Materials*

# A Simple Cerium Coating Strategy for Titanium Oxide Nanotubes' Bioactivity Enhancement

**Serena De Santis [1,*], Giovanni Sotgiu [1], Francesco Porcelli [1], Martina Marsotto [2], Giovanna Iucci [2] and Monica Orsini [1]**

[1] Department of Engineering, Roma Tre University, via Vito Volterra 62, 00146 Rome, Italy; giovanni.sotgiu@uniroma3.it (G.S.); francesco.porcelli@uniroma3.it (F.P.); monica.orsini@uniroma3.it (M.O.)
[2] Department of Science, Roma Tre University, Via della Vasca Navale 79, 00146 Rome, Italy; martina.marsotto@uniroma3.it (M.M.); giovanna.iucci@uniroma3.it (G.I.)
* Correspondence: serena.desantis@uniroma3.it

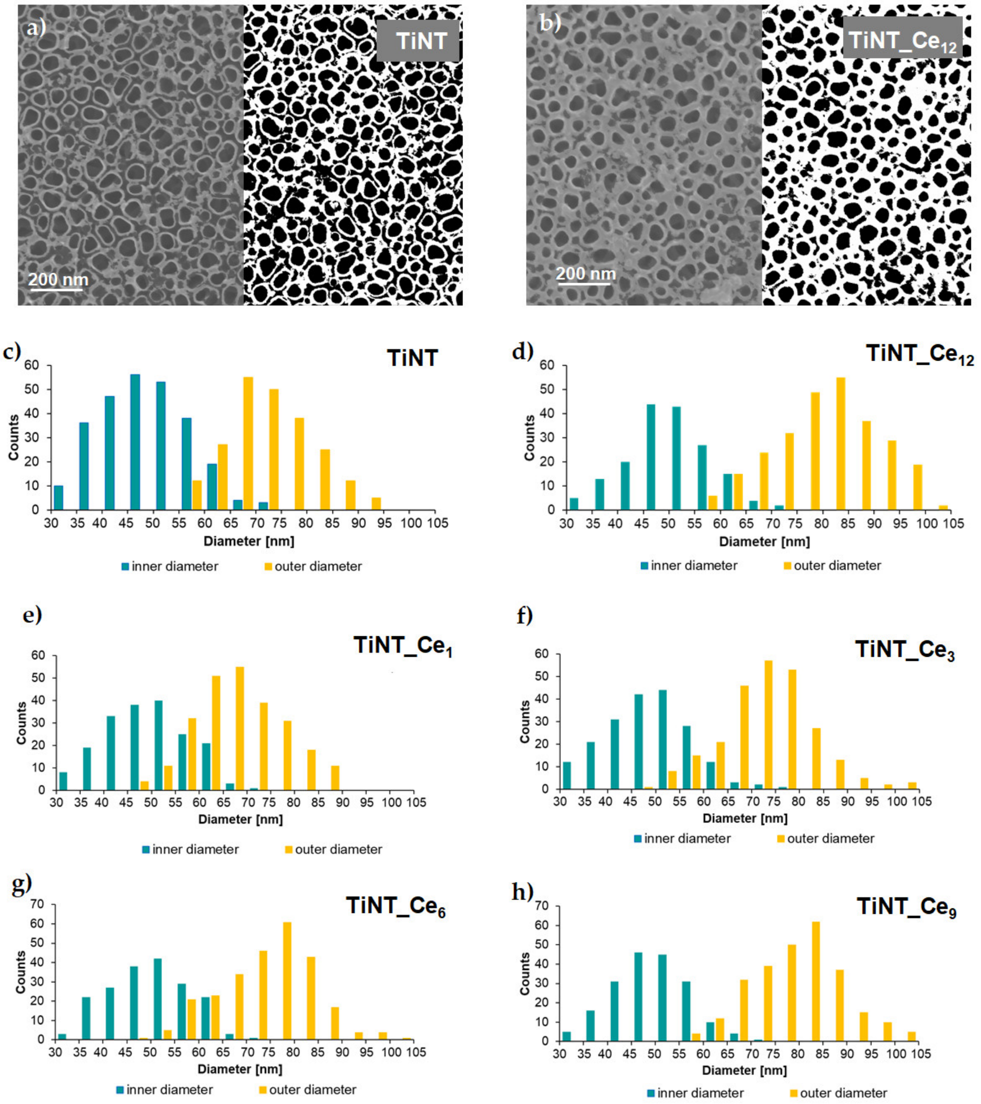


**Figure S1.** Image analysis of the FE-SEM micrographies for TiNT and TiNT_Ce$_n$. Panels (**a**) and (**b**) show the original SEM output (in gray) partially superimposed to the binarized image generate for the calculation. Panel (**c**–**h**) report the inner and outer diameter distribution for each sample.

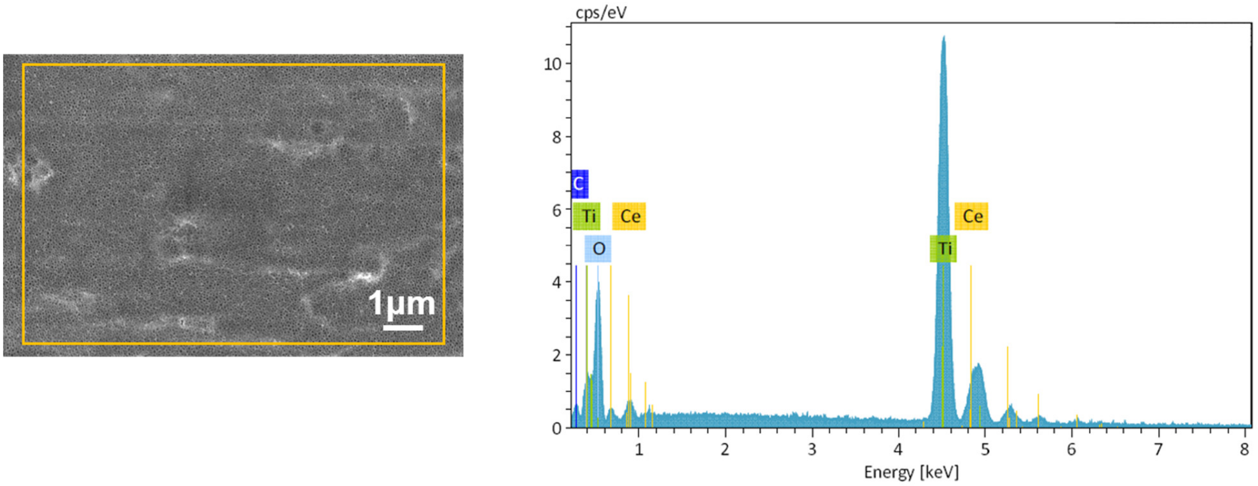


**Figure S2.** FE-SEM micrograph and EDS spectrum relative to the selected area. EDS were performed at 15 kV at the same magnification and corresponding total area for every sample.

**Table S1.** Elemental composition (in atomic %) of TiNT and TiNT_Cen samples.

| | Ti | O | Ce | C |
|---|---|---|---|---|
| TiNT | 71.52 | 26.76 | - | 1.72 |
| TiNT_Ce$_1$ | 70.76 | 25.34 | 0.8 | 3.10 |
| TiNT_Ce$_3$ | 68.59 | 27.21 | 2.37 | 1.83 |
| TiNT_Ce$_6$ | 66.39 | 28.73 | 4.06 | 0.82 |
| TiNT_Ce$_9$ | 64.67 | 26.97 | 6.95 | 1.41 |
| TiNT_Ce$_{12}$ | 61.49 | 29.22 | 8.11 | 1.18 |

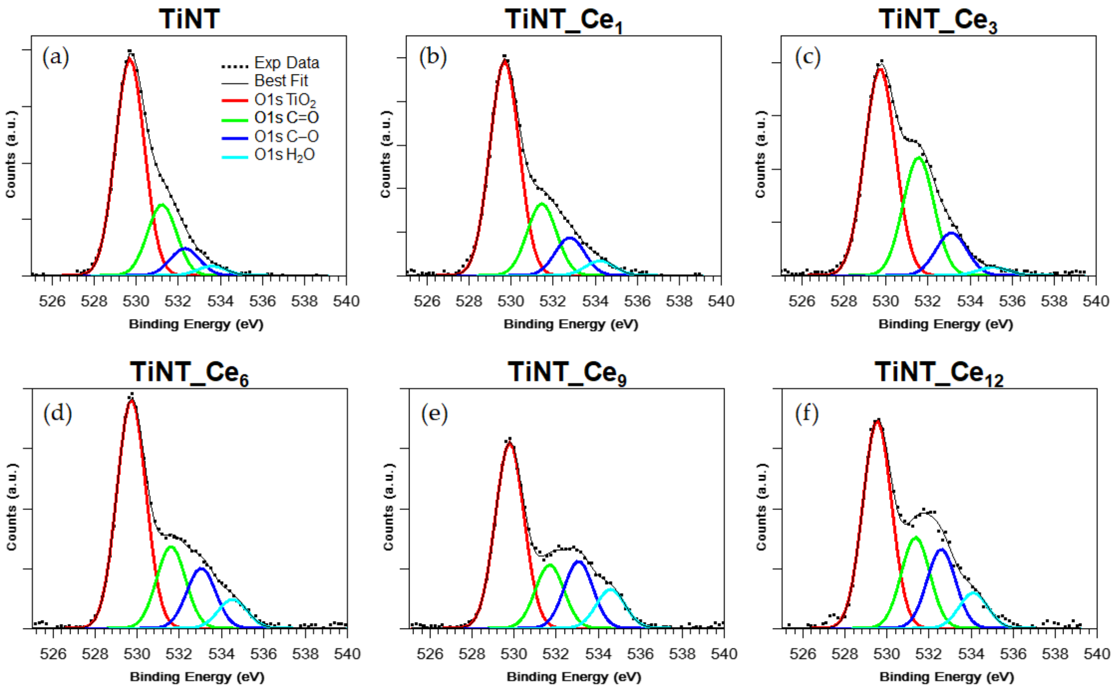


**Figure S3.** XPS O1s fitted spectra for (**a**) TiNT and (**b**–**f**) TiNT_Cen samples. Data were fitted with four components of about 530, 531.5, 533 and 534 eV that correspond to $O^{2-}$ of metal oxides, carbonyl oxygens, always present in samples prepared in air, hydroxyl groups (or chemisorbed water) and physisorbed water, respectively.

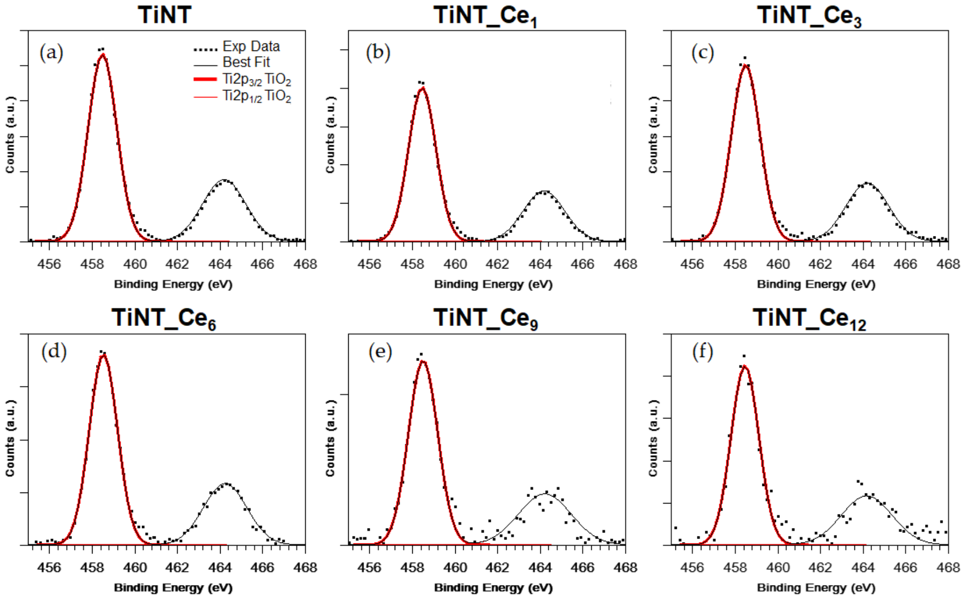


**Figure S4.** XPS Ti2p fitted spectra for (**a**) TiNT and (**b**–**f**) TiNT_Ce$_n$ samples.

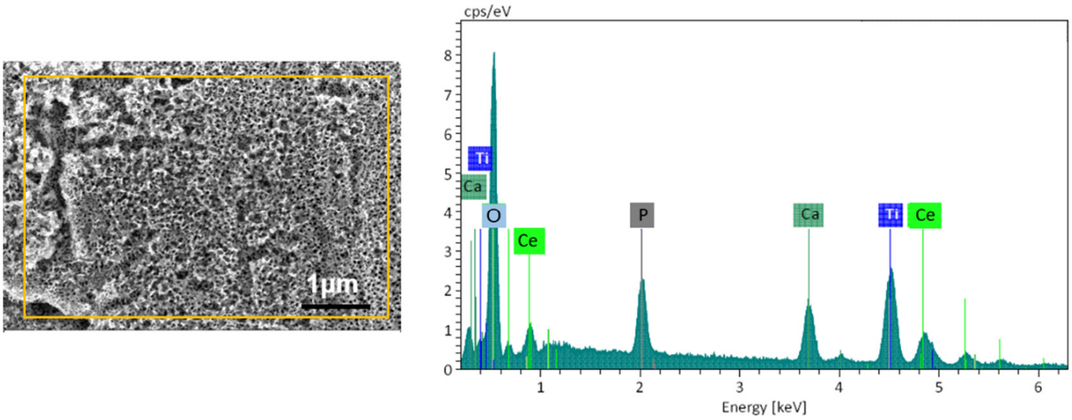


**Figure S5.** FE-SEM micrograph and EDS spectrum relative to the selected area. EDS were performed at 15 kV at the same magnification and corresponding total area for every sample.

**Table S2.** Elemental composition (in atomic %) of TiNT and TiNT_Ce$_n$ samples after 8 days soaking in SBF at 37 °C.

| | Ca | P | Ca/P |
|---|---|---|---|
| TiNT | - | - | - |
| TiNT_Ce$_1$ | 0.60 | 0.42 | 1.42 |
| TiNT_Ce$_3$ | 1.36 | 0.91 | 1.49 |
| TiNT_Ce$_6$ | 2.39 | 1.57 | 1.52 |
| TiNT_Ce$_9$ | 4.33 | 2.87 | 1.51 |
| TiNT_Ce$_{12}$ | 4.77 | 3.04 | 1.57 |
